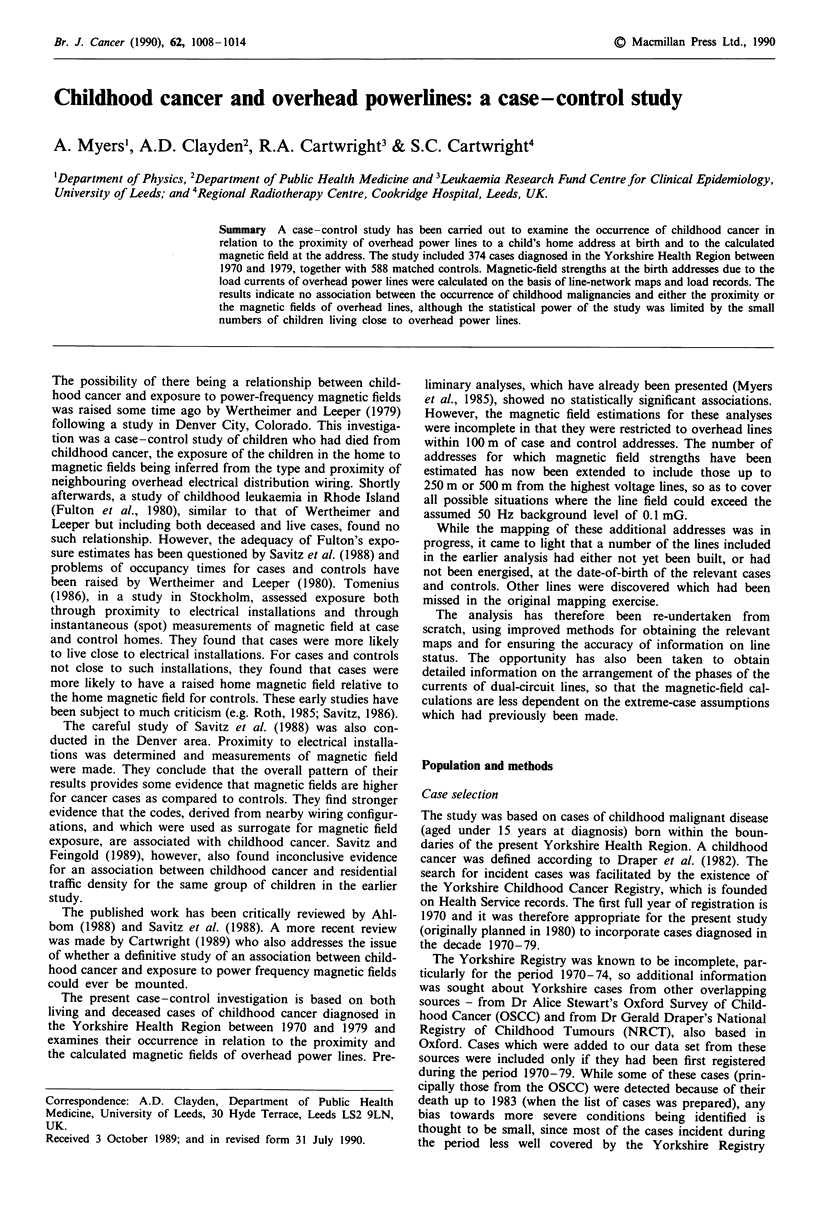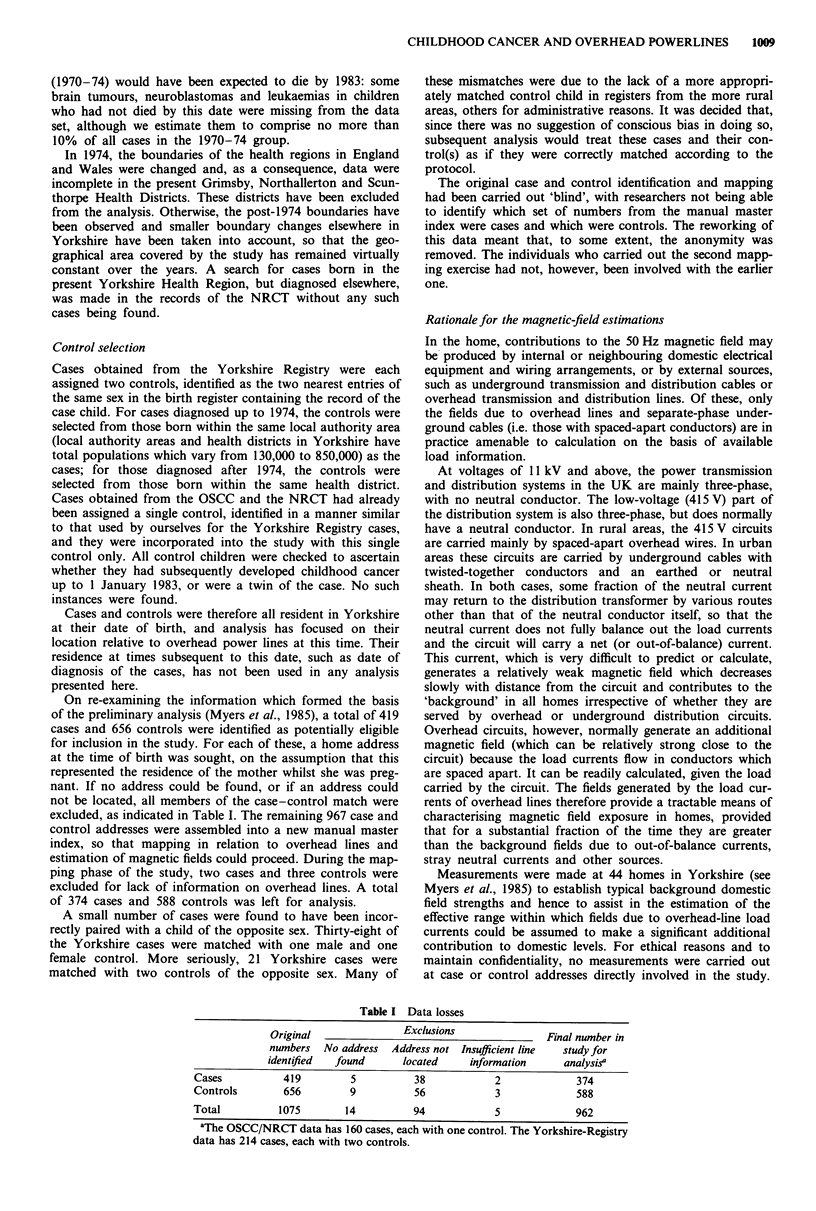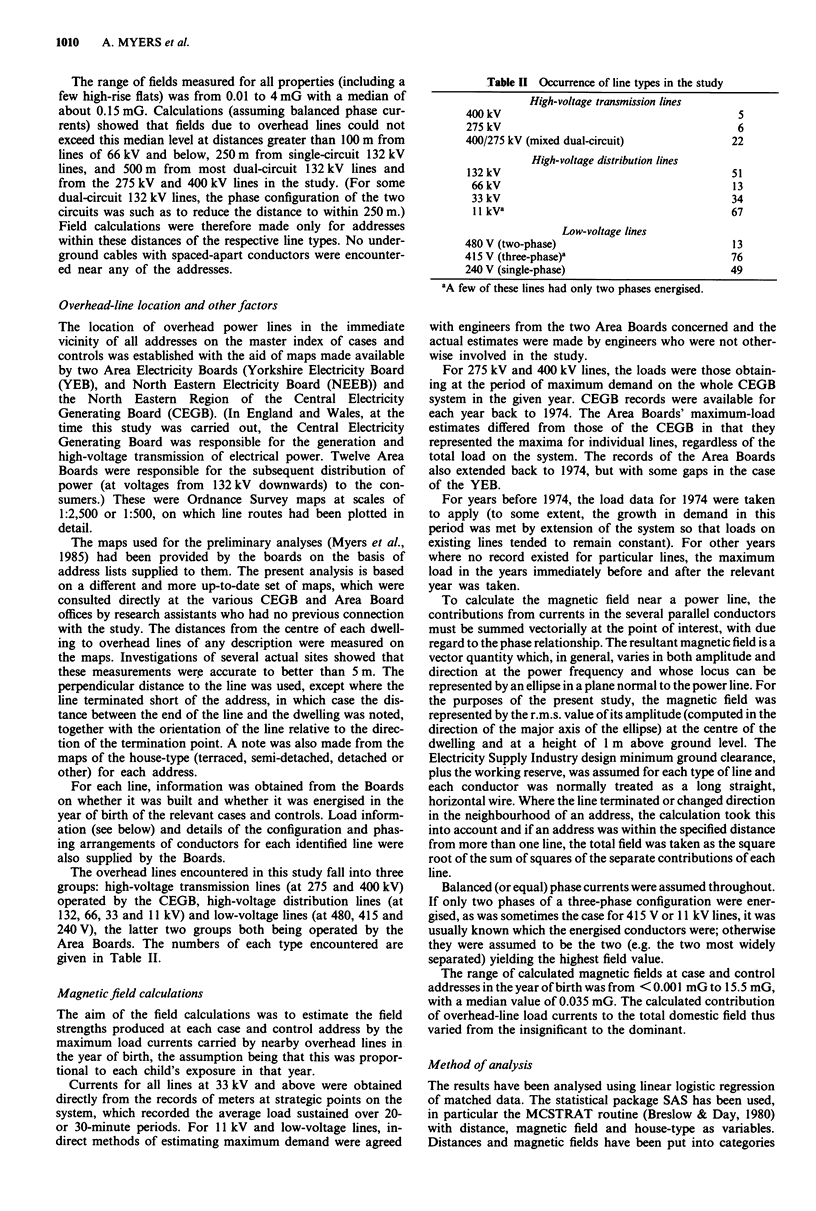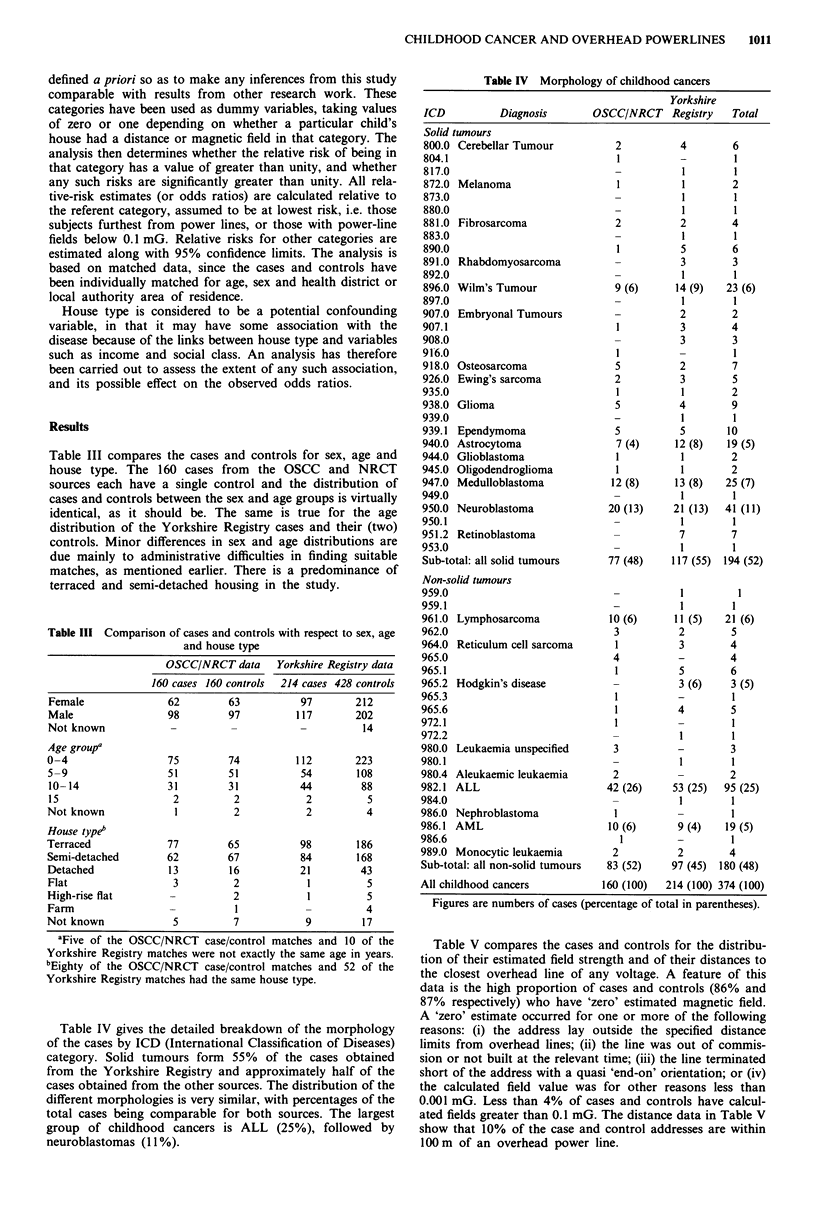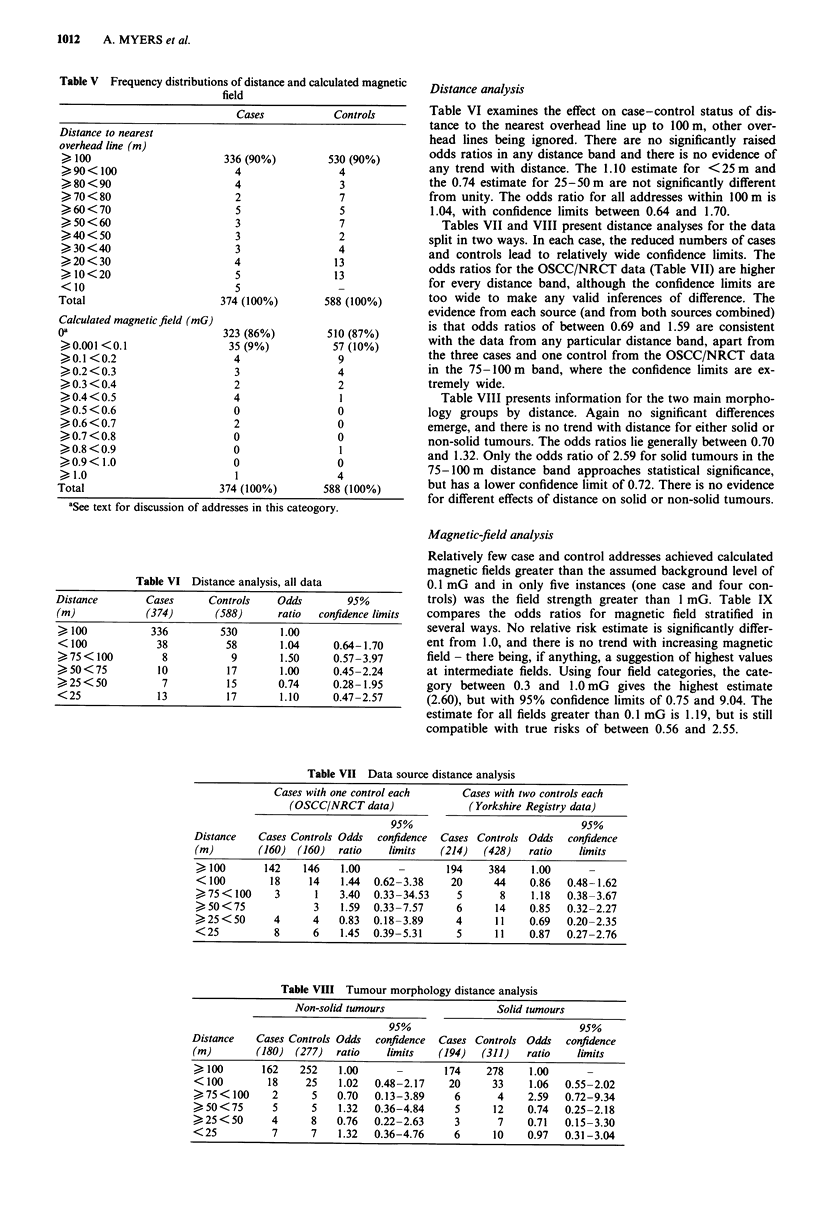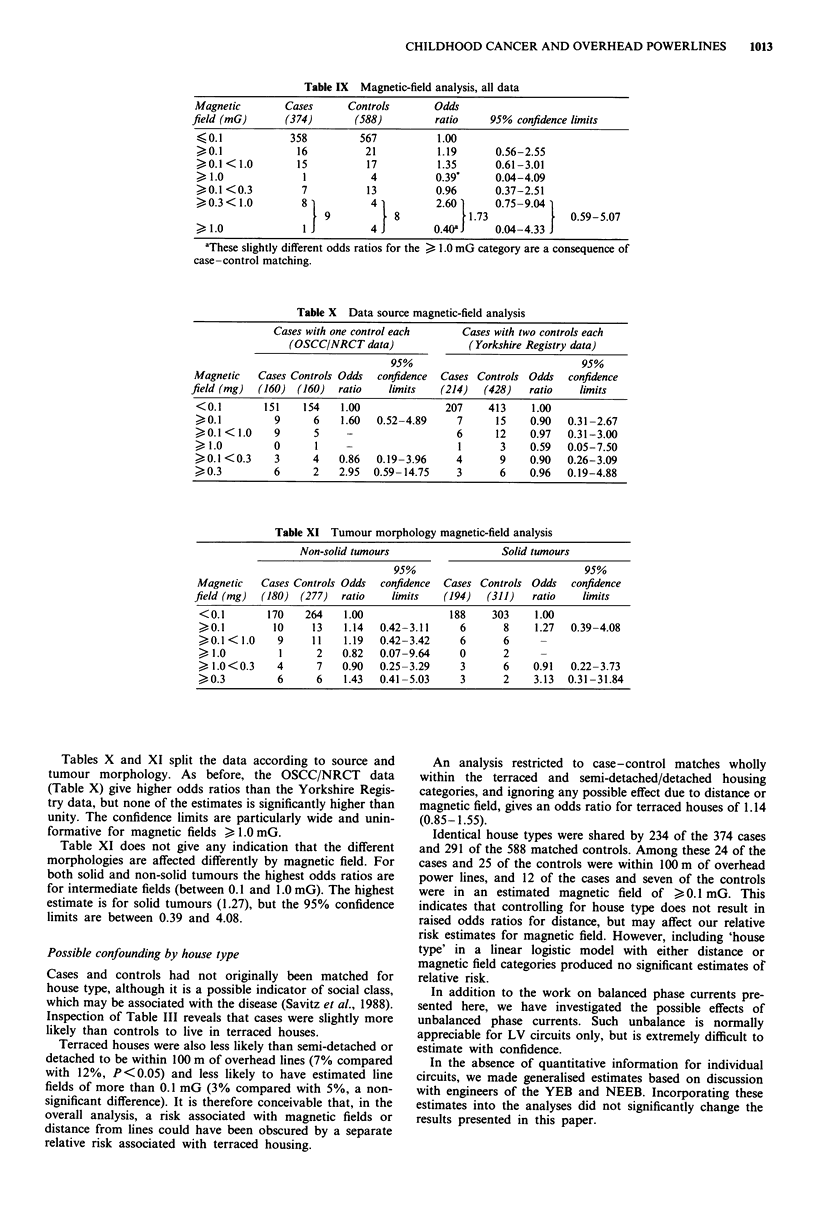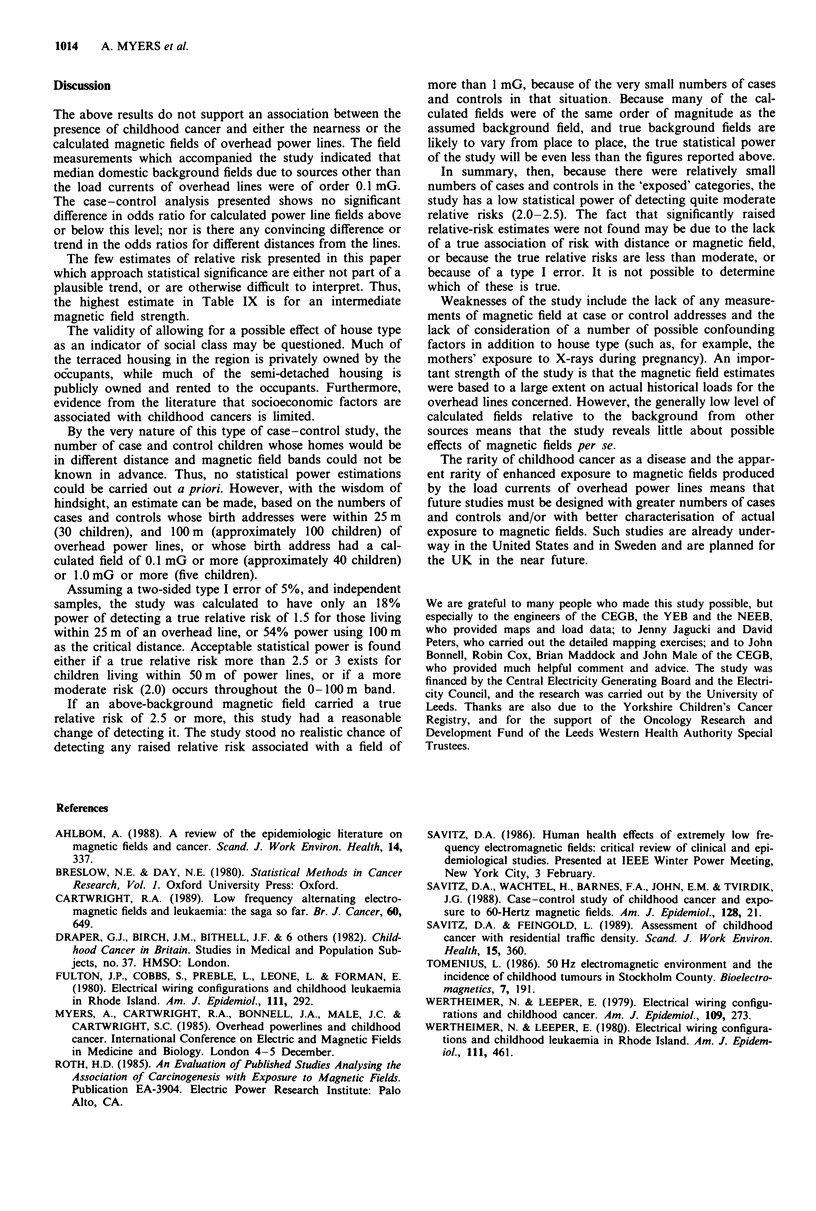# Childhood cancer and overhead powerlines: a case-control study.

**DOI:** 10.1038/bjc.1990.428

**Published:** 1990-12

**Authors:** A. Myers, A. D. Clayden, R. A. Cartwright, S. C. Cartwright

**Affiliations:** Department of Physics, University of Leeds, UK.

## Abstract

A case-control study has been carried out to examine the occurrence of childhood cancer in relation to the proximity of overhead power lines to a child's home address at birth and to the calculated magnetic field at the address. The study included 374 cases diagnosed in the Yorkshire Health Region between 1970 and 1979, together with 588 matched controls. Magnetic-field strengths at the birth addresses due to the load currents of overhead power lines were calculated on the basis of line-network maps and load records. The results indicate no association between the occurrence of childhood malignancies and either the proximity or the magnetic fields of overhead lines, although the statistical power of the study was limited by the small numbers of children living close to overhead power lines.


*Br. J. Cancer* (1990), **62**, 1008–1014 © Macmillan Press Ltd., 1990

### Childhood cancer and overhead powerlines: a case–control study

A. Myers[1], A.D. Clayden[2], R.A. Cartwright[3] & S.C. Cartwright[4]

[1]*Department of Physics,* [2]*Department of Public Health Medicine and* [3]*Leukaemia Research Fund Centre for Clinical Epidemiology, University of Leeds; and* [4]*Regional Radiotherapy Centre, Cookridge Hospital, Leeds, UK.*

**Summary** A case–control study has been carried out to examine the occurrence of childhood cancer in relation to the proximity of overhead power lines to a child's home address at birth and to the calculated magnetic field at the address. The study included 374 cases diagnosed in the Yorkshire Health Region between 1970 and 1979, together with 588 matched controls. Magnetic-field strengths at the birth addresses due to the load currents of overhead power lines were calculated on the basis of line-network maps and load records. The results indicate no association between the occurrence of childhood malignancies and either the proximity or the magnetic fields of overhead lines, although the statistical power of the study was limited by the small numbers of children living close to overhead power lines.

The possibility of there being a relationship between childhood cancer and exposure to power-frequency magnetic fields was raised some time ago by Wertheimer and Leeper (1979) following a study in Denver City, Colorado. This investigation was a case–control study of children who had died from childhood cancer, the exposure of the children in the home to magnetic fields being inferred from the type and proximity of neighbouring overhead electrical distribution wiring. Shortly afterwards, a study of childhood leukaemia in Rhode Island (Fulton *et al.*, 1980), similar to that of Wertheimer and Leeper but including both deceased and live cases, found no such relationship. However, the adequacy of Fulton's exposure estimates has been questioned by Savitz *et al.* (1988) and problems of occupancy times for cases and controls have been raised by Wertheimer and Leeper (1980). Tomenius (1986), in a study in Stockholm, assessed exposure both through proximity to electrical installations and through instantaneous (spot) measurements of magnetic field at case and control homes. They found that cases were more likely to live close to electrical installations. For cases and controls not close to such installations, they found that cases were more likely to have a raised home magnetic field relative to the home magnetic field for controls. These early studies have been subject to much criticism (e.g. Roth, 1985; Savitz, 1986).

The careful study of Savitz *et al.* (1988) was also conducted in the Denver area. Proximity to electrical installations was determined and measurements of magnetic field were made. They conclude that the overall pattern of their results provides some evidence that magnetic fields are higher for cancer cases as compared to controls. They find stronger evidence that the codes, derived from nearby wiring configurations, and which were used as surrogate for magnetic field exposure, are associated with childhood cancer. Savitz and Feingold (1989), however, also found inconclusive evidence for an association between childhood cancer and residential traffic density for the same group of children in the earlier study.

The published work has been critically reviewed by Ahlbom (1988) and Savitz *et al.* (1988). A more recent review was made by Cartwright (1989) who also addresses the issue of whether a definitive study of an association between childhood cancer and exposure to power frequency magnetic fields could ever be mounted.

The present case–control investigation is based on both living and deceased cases of childhood cancer diagnosed in the Yorkshire Health Region between 1970 and 1979 and examines their occurrence in relation to the proximity and the calculated magnetic fields of overhead power lines. Preliminary analyses, which have already been presented (Myers *et al.*, 1985), showed no statistically significant associations. However, the magnetic field estimations for these analyses were incomplete in that they were restricted to overhead lines within 100 m of case and control addresses. The number of addresses for which magnetic field strengths have been estimated has now been extended to include those up to 250 m or 500 m from the highest voltage lines, so as to cover all possible situations where the line field could exceed the assumed 50 Hz background level of 0.1 mG.

While the mapping of these additional addresses was in progress, it came to light that a number of the lines included in the earlier analysis had either not yet been built, or had not been energised, at the date-of-birth of the relevant cases and controls. Other lines were discovered which had been missed in the original mapping exercise.

The analysis has therefore been re-undertaken from scratch, using improved methods for obtaining the relevant maps and for ensuring the accuracy of information on line status. The opportunity has also been taken to obtain detailed information on the arrangement of the phases of the currents of dual-circuit lines, so that the magnetic-field calculations are less dependent on the extreme-case assumptions which had previously been made.

### Population and methods

### *Case selection*

The study was based on cases of childhood malignant disease (aged under 15 years at diagnosis) born within the boundaries of the present Yorkshire Health Region. A childhood cancer was defined according to Draper *et al.* (1982). The search for incident cases was facilitated by the existence of the Yorkshire Childhood Cancer Registry, which is founded on Health Service records. The first full year of registration is 1970 and it was therefore appropriate for the present study (originally planned in 1980) to incorporate cases diagnosed in the decade 1970–79.

The Yorkshire Registry was known to be incomplete, particularly for the period 1970–74, so additional information was sought about Yorkshire cases from other overlapping sources – from Dr Alice Stewart's Oxford Survey of Childhood Cancer (OSCC) and from Dr Gerald Draper's National Registry of Childhood Tumours (NRCT), also based in Oxford. Cases which were added to our data set from these sources were included only if they had been first registered during the period 1970–79. While some of these cases (principally those from the OSCC) were detected because of their death up to 1983 (when the list of cases was prepared), any bias towards more severe conditions being identified is thought to be small, since most of the cases incident during the period less well covered by the Yorkshire Registry

Correspondence: A.D. Clayden, Department of Public Health Medicine, University of Leeds, 30 Hyde Terrace, Leeds LS2 9LN, UK.
Received 3 October 1989; and in revised form 31 July 1990.

(1970–74) would have been expected to die by 1983: some brain tumours, neuroblastomas and leukaemias in children who had not died by this date were missing from the data set, although we estimate them to comprise no more than 10% of all cases in the 1970–74 group.

In 1974, the boundaries of the health regions in England and Wales were changed and, as a consequence, data were incomplete in the present Grimsby, Northallerton and Scunthorpe Health Districts. These districts have been excluded from the analysis. Otherwise, the post-1974 boundaries have been observed and smaller boundary changes elsewhere in Yorkshire have been taken into account, so that the geographical area covered by the study has remained virtually constant over the years. A search for cases born in the present Yorkshire Health Region, but diagnosed elsewhere, was made in the records of the NRCT without any such cases being found.

*Control selection*

Cases obtained from the Yorkshire Registry were each assigned two controls, identified as the two nearest entries of the same sex in the birth register containing the record of the case child. For cases diagnosed up to 1974, the controls were selected from those born within the same local authority area (local authority areas and health districts in Yorkshire have total populations which vary from 130,000 to 850,000) as the cases; for those diagnosed after 1974, the controls were selected from those born within the same health district. Cases obtained from the OSCC and the NRCT had already been assigned a single control, identified in a manner similar to that used by ourselves for the Yorkshire Registry cases, and they were incorporated into the study with this single control only. All control children were checked to ascertain whether they had subsequently developed childhood cancer up to 1 January 1983, or were a twin of the case. No such instances were found.

Cases and controls were therefore all resident in Yorkshire at their date of birth, and analysis has focused on their location relative to overhead power lines at this time. Their residence at times subsequent to this date, such as date of diagnosis of the cases, has not been used in any analysis presented here.

On re-examining the information which formed the basis of the preliminary analysis (Myers *et al.*, 1985), a total of 419 cases and 656 controls were identified as potentially eligible for inclusion in the study. For each of these, a home address at the time of birth was sought, on the assumption that this represented the residence of the mother whilst she was pregnant. If no address could be found, or if an address could not be located, all members of the case–control match were excluded, as indicated in Table I. The remaining 967 case and control addresses were assembled into a new manual master index, so that mapping in relation to overhead lines and estimation of magnetic fields could proceed. During the mapping phase of the study, two cases and three controls were excluded for lack of information on overhead lines. A total of 374 cases and 588 controls was left for analysis.

A small number of cases were found to have been incorrectly paired with a child of the opposite sex. Thirty-eight of the Yorkshire cases were matched with one male and one female control. More seriously, 21 Yorkshire cases were matched with two controls of the opposite sex. Many of these mismatches were due to the lack of a more appropriately matched control child in registers from the more rural areas, others for administrative reasons. It was decided that, since there was no suggestion of conscious bias in doing so, subsequent analysis would treat these cases and their control(s) as if they were correctly matched according to the protocol.

The original case and control identification and mapping had been carried out 'blind', with researchers not being able to identify which set of numbers from the manual master index were cases and which were controls. The reworking of this data meant that, to some extent, the anonymity was removed. The individuals who carried out the second mapping exercise had not, however, been involved with the earlier one.

*Rationale for the magnetic-field estimations*

In the home, contributions to the 50 Hz magnetic field may be produced by internal or neighbouring domestic electrical equipment and wiring arrangements, or by external sources, such as underground transmission and distribution cables or overhead transmission and distribution lines. Of these, only the fields due to overhead lines and separate-phase underground cables (i.e. those with spaced-apart conductors) are in practice amenable to calculation on the basis of available load information.

At voltages of 11 kV and above, the power transmission and distribution systems in the UK are mainly three-phase, with no neutral conductor. The low-voltage (415 V) part of the distribution system is also three-phase, but does normally have a neutral conductor. In rural areas, the 415 V circuits are carried mainly by spaced-apart overhead wires. In urban areas these circuits are carried by underground cables with twisted-together conductors and an earthed or neutral sheath. In both cases, some fraction of the neutral current may return to the distribution transformer by various routes other than that of the neutral conductor itself, so that the neutral current does not fully balance out the load currents and the circuit will carry a net (or out-of-balance) current. This current, which is very difficult to predict or calculate, generates a relatively weak magnetic field which decreases slowly with distance from the circuit and contributes to the 'background' in all homes irrespective of whether they are served by overhead or underground distribution circuits. Overhead circuits, however, normally generate an additional magnetic field (which can be relatively strong close to the circuit) because the load currents flow in conductors which are spaced apart. It can be readily calculated, given the load carried by the circuit. The fields generated by the load currents of overhead lines therefore provide a tractable means of characterising magnetic field exposure in homes, provided that for a substantial fraction of the time they are greater than the background fields due to out-of-balance currents, stray neutral currents and other sources.

Measurements were made at 44 homes in Yorkshire (see Myers *et al.*, 1985) to establish typical background domestic field strengths and hence to assist in the estimation of the effective range within which fields due to overhead-line load currents could be assumed to make a significant additional contribution to domestic levels. For ethical reasons and to maintain confidentiality, no measurements were carried out at case or control addresses directly involved in the study.

**Table I** Data losses

| | Original numbers identified | Exclusions: No address found | Exclusions: Address not located | Exclusions: Insufficient line information | Final number in study for analysis[a] |
|---|---|---|---|---|---|
| Cases | 419 | 5 | 38 | 2 | 374 |
| Controls | 656 | 9 | 56 | 3 | 588 |
| Total | 1075 | 14 | 94 | 5 | 962 |

[a]The OSCC/NRCT data has 160 cases, each with one control. The Yorkshire-Registry data has 214 cases, each with two controls.

The range of fields measured for all properties (including a few high-rise flats) was from 0.01 to 4 mG with a median of about 0.15 mG. Calculations (assuming balanced phase currents) showed that fields due to overhead lines could not exceed this median level at distances greater than 100 m from lines of 66 kV and below, 250 m from single-circuit 132 kV lines, and 500 m from most dual-circuit 132 kV lines and from the 275 kV and 400 kV lines in the study. (For some dual-circuit 132 kV lines, the phase configuration of the two circuits was such as to reduce the distance to within 250 m.) Field calculations were therefore made only for addresses within these distances of the respective line types. No underground cables with spaced-apart conductors were encountered near any of the addresses.

Table II Occurrence of line types in the study

| | |
|---|---|
| *High-voltage transmission lines* | |
| 400 kV | 5 |
| 275 kV | 6 |
| 400/275 kV (mixed dual-circuit) | 22 |
| *High-voltage distribution lines* | |
| 132 kV | 51 |
| 66 kV | 13 |
| 33 kV | 34 |
| 11 kV[a] | 67 |
| *Low-voltage lines* | |
| 480 V (two-phase) | 13 |
| 415 V (three-phase)[a] | 76 |
| 240 V (single-phase) | 49 |

[a]A few of these lines had only two phases energised.

*Overhead-line location and other factors*

The location of overhead power lines in the immediate vicinity of all addresses on the master index of cases and controls was established with the aid of maps made available by two Area Electricity Boards (Yorkshire Electricity Board (YEB), and North Eastern Electricity Board (NEEB)) and the North Eastern Region of the Central Electricity Generating Board (CEGB). (In England and Wales, at the time this study was carried out, the Central Electricity Generating Board was responsible for the generation and high-voltage transmission of electrical power. Twelve Area Boards were responsible for the subsequent distribution of power (at voltages from 132 kV downwards) to the consumers.) These were Ordnance Survey maps at scales of 1:2,500 or 1:500, on which line routes had been plotted in detail.

The maps used for the preliminary analyses (Myers *et al.*, 1985) had been provided by the boards on the basis of address lists supplied to them. The present analysis is based on a different and more up-to-date set of maps, which were consulted directly at the various CEGB and Area Board offices by research assistants who had no previous connection with the study. The distances from the centre of each dwelling to overhead lines of any description were measured on the maps. Investigations of several actual sites showed that these measurements were accurate to better than 5 m. The perpendicular distance to the line was used, except where the line terminated short of the address, in which case the distance between the end of the line and the dwelling was noted, together with the orientation of the line relative to the direction of the termination point. A note was also made from the maps of the house-type (terraced, semi-detached, detached or other) for each address.

For each line, information was obtained from the Boards on whether it was built and whether it was energised in the year of birth of the relevant cases and controls. Load information (see below) and details of the configuration and phasing arrangements of conductors for each identified line were also supplied by the Boards.

The overhead lines encountered in this study fall into three groups: high-voltage transmission lines (at 275 and 400 kV) operated by the CEGB, high-voltage distribution lines (at 132, 66, 33 and 11 kV) and low-voltage lines (at 480, 415 and 240 V), the latter two groups both being operated by the Area Boards. The numbers of each type encountered are given in Table II.

*Magnetic field calculations*

The aim of the field calculations was to estimate the field strengths produced at each case and control address by the maximum load currents carried by nearby overhead lines in the year of birth, the assumption being that this was proportional to each child's exposure in that year.

Currents for all lines at 33 kV and above were obtained directly from the records of meters at strategic points on the system, which recorded the average load sustained over 20- or 30-minute periods. For 11 kV and low-voltage lines, indirect methods of estimating maximum demand were agreed with engineers from the two Area Boards concerned and the actual estimates were made by engineers who were not otherwise involved in the study.

For 275 kV and 400 kV lines, the loads were those obtaining at the period of maximum demand on the whole CEGB system in the given year. CEGB records were available for each year back to 1974. The Area Boards' maximum-load estimates differed from those of the CEGB in that they represented the maxima for individual lines, regardless of the total load on the system. The records of the Area Boards also extended back to 1974, but with some gaps in the case of the YEB.

For years before 1974, the load data for 1974 were taken to apply (to some extent, the growth in demand in this period was met by extension of the system so that loads on existing lines tended to remain constant). For other years where no record existed for particular lines, the maximum load in the years immediately before and after the relevant year was taken.

To calculate the magnetic field near a power line, the contributions from currents in the several parallel conductors must be summed vectorially at the point of interest, with due regard to the phase relationship. The resultant magnetic field is a vector quantity which, in general, varies in both amplitude and direction at the power frequency and whose locus can be represented by an ellipse in a plane normal to the power line. For the purposes of the present study, the magnetic field was represented by the r.m.s. value of its amplitude (computed in the direction of the major axis of the ellipse) at the centre of the dwelling and at a height of 1 m above ground level. The Electricity Supply Industry design minimum ground clearance, plus the working reserve, was assumed for each type of line and each conductor was normally treated as a long straight, horizontal wire. Where the line terminated or changed direction in the neighbourhood of an address, the calculation took this into account and if an address was within the specified distance from more than one line, the total field was taken as the square root of the sum of squares of the separate contributions of each line.

Balanced (or equal) phase currents were assumed throughout. If only two phases of a three-phase configuration were energised, as was sometimes the case for 415 V or 11 kV lines, it was usually known which the energised conductors were; otherwise they were assumed to be the two (e.g. the two most widely separated) yielding the highest field value.

The range of calculated magnetic fields at case and control addresses in the year of birth was from $<0.001$ mG to 15.5 mG, with a median value of 0.035 mG. The calculated contribution of overhead-line load currents to the total domestic field thus varied from the insignificant to the dominant.

*Method of analysis*

The results have been analysed using linear logistic regression of matched data. The statistical package SAS has been used, in particular the MCSTRAT routine (Breslow & Day, 1980) with distance, magnetic field and house-type as variables. Distances and magnetic fields have been put into categories defined *a priori* so as to make any inferences from this study comparable with results from other research work. These categories have been used as dummy variables, taking values of zero or one depending on whether a particular child's house had a distance or magnetic field in that category. The analysis then determines whether the relative risk of being in that category has a value of greater than unity, and whether any such risks are significantly greater than unity. All relative-risk estimates (or odds ratios) are calculated relative to the referent category, assumed to be at lowest risk, i.e. those subjects furthest from power lines, or those with power-line fields below 0.1 mG. Relative risks for other categories are estimated along with 95% confidence limits. The analysis is based on matched data, since the cases and controls have been individually matched for age, sex and health district or local authority area of residence.

House type is considered to be a potential confounding variable, in that it may have some association with the disease because of the links between house type and variables such as income and social class. An analysis has therefore been carried out to assess the extent of any such association, and its possible effect on the observed odds ratios.

### Results

Table III compares the cases and controls for sex, age and house type. The 160 cases from the OSCC and NRCT sources each have a single control and the distribution of cases and controls between the sex and age groups is virtually identical, as it should be. The same is true for the age distribution of the Yorkshire Registry cases and their (two) controls. Minor differences in sex and age distributions are due mainly to administrative difficulties in finding suitable matches, as mentioned earlier. There is a predominance of terraced and semi-detached housing in the study.

**Table III** Comparison of cases and controls with respect to sex, age and house type

| | *OSCC/NRCT data* | | *Yorkshire Registry data* | |
|---|---|---|---|---|
| | *160 cases* | *160 controls* | *214 cases* | *428 controls* |
| Female | 62 | 63 | 97 | 212 |
| Male | 98 | 97 | 117 | 202 |
| Not known | – | – | – | 14 |
| *Age group*[a] | | | | |
| 0–4 | 75 | 74 | 112 | 223 |
| 5–9 | 51 | 51 | 54 | 108 |
| 10–14 | 31 | 31 | 44 | 88 |
| 15 | 2 | 2 | 2 | 5 |
| Not known | 1 | 2 | 2 | 4 |
| *House type*[b] | | | | |
| Terraced | 77 | 65 | 98 | 186 |
| Semi-detached | 62 | 67 | 84 | 168 |
| Detached | 13 | 16 | 21 | 43 |
| Flat | 3 | 2 | 1 | 5 |
| High-rise flat | – | 2 | 1 | 5 |
| Farm | – | 1 | – | 4 |
| Not known | 5 | 7 | 9 | 17 |

[a]Five of the OSCC/NRCT case/control matches and 10 of the Yorkshire Registry matches were not exactly the same age in years. [b]Eighty of the OSCC/NRCT case/control matches and 52 of the Yorkshire Registry matches had the same house type.

Table IV gives the detailed breakdown of the morphology of the cases by ICD (International Classification of Diseases) category. Solid tumours form 55% of the cases obtained from the Yorkshire Registry and approximately half of the cases obtained from the other sources. The distribution of the different morphologies is very similar, with percentages of the total cases being comparable for both sources. The largest group of childhood cancers is ALL (25%), followed by neuroblastomas (11%).

**Table IV** Morphology of childhood cancers

| *ICD* | *Diagnosis* | *OSCC/NRCT* | *Yorkshire Registry* | *Total* |
|---|---|---|---|---|
| *Solid tumours* | | | | |
| 800.0 | Cerebellar Tumour | 2 | 4 | 6 |
| 804.1 | | 1 | – | 1 |
| 817.0 | | – | 1 | 1 |
| 872.0 | Melanoma | 1 | 1 | 2 |
| 873.0 | | – | 1 | 1 |
| 880.0 | | – | 1 | 1 |
| 881.0 | Fibrosarcoma | 2 | 2 | 4 |
| 883.0 | | – | 1 | 1 |
| 890.0 | | 1 | 5 | 6 |
| 891.0 | Rhabdomyosarcoma | – | 3 | 3 |
| 892.0 | | – | 1 | 1 |
| 896.0 | Wilm's Tumour | 9 (6) | 14 (9) | 23 (6) |
| 897.0 | | – | 1 | 1 |
| 907.0 | Embryonal Tumours | – | 2 | 2 |
| 907.1 | | 1 | 3 | 4 |
| 908.0 | | – | 3 | 3 |
| 916.0 | | 1 | – | 1 |
| 918.0 | Osteosarcoma | 5 | 2 | 7 |
| 926.0 | Ewing's sarcoma | 2 | 3 | 5 |
| 935.0 | | 1 | 1 | 2 |
| 938.0 | Glioma | 5 | 4 | 9 |
| 939.0 | | – | 1 | 1 |
| 939.1 | Ependymoma | 5 | 5 | 10 |
| 940.0 | Astrocytoma | 7 (4) | 12 (8) | 19 (5) |
| 944.0 | Glioblastoma | 1 | 1 | 2 |
| 945.0 | Oligodendroglioma | 1 | 1 | 2 |
| 947.0 | Medulloblastoma | 12 (8) | 13 (8) | 25 (7) |
| 949.0 | | – | 1 | 1 |
| 950.0 | Neuroblastoma | 20 (13) | 21 (13) | 41 (11) |
| 950.1 | | – | 1 | 1 |
| 951.2 | Retinoblastoma | – | 7 | 7 |
| 953.0 | | – | 1 | 1 |
| Sub-total: all solid tumours | | 77 (48) | 117 (55) | 194 (52) |
| *Non-solid tumours* | | | | |
| 959.0 | | – | 1 | 1 |
| 959.1 | | – | 1 | 1 |
| 961.0 | Lymphosarcoma | 10 (6) | 11 (5) | 21 (6) |
| 962.0 | | 3 | 2 | 5 |
| 964.0 | Reticulum cell sarcoma | 1 | 3 | 4 |
| 965.0 | | 4 | – | 4 |
| 965.1 | | 1 | 5 | 6 |
| 965.2 | Hodgkin's disease | – | 3 (6) | 3 (5) |
| 965.3 | | 1 | – | 1 |
| 965.6 | | 1 | 4 | 5 |
| 972.1 | | 1 | – | 1 |
| 972.2 | | – | 1 | 1 |
| 980.0 | Leukaemia unspecified | 3 | – | 3 |
| 980.1 | | – | 1 | 1 |
| 980.4 | Aleukaemic leukaemia | 2 | – | 2 |
| 982.1 | ALL | 42 (26) | 53 (25) | 95 (25) |
| 984.0 | | – | 1 | 1 |
| 986.0 | Nephroblastoma | 1 | – | 1 |
| 986.1 | AML | 10 (6) | 9 (4) | 19 (5) |
| 986.6 | | 1 | – | 1 |
| 989.0 | Monocytic leukaemia | 2 | 2 | 4 |
| Sub-total: all non-solid tumours | | 83 (52) | 97 (45) | 180 (48) |
| All childhood cancers | | 160 (100) | 214 (100) | 374 (100) |

Figures are numbers of cases (percentage of total in parentheses).

Table V compares the cases and controls for the distribution of their estimated field strength and of their distances to the closest overhead line of any voltage. A feature of this data is the high proportion of cases and controls (86% and 87% respectively) who have 'zero' estimated magnetic field. A 'zero' estimate occurred for one or more of the following reasons: (i) the address lay outside the specified distance limits from overhead lines; (ii) the line was out of commission or not built at the relevant time; (iii) the line terminated short of the address with a quasi 'end-on' orientation; or (iv) the calculated field value was for other reasons less than 0.001 mG. Less than 4% of cases and controls have calculated fields greater than 0.1 mG. The distance data in Table V show that 10% of the case and control addresses are within 100 m of an overhead power line.

**Table V** Frequency distributions of distance and calculated magnetic field

| | Cases | Controls |
|---|---|---|
| *Distance to nearest overhead line (m)* | | |
| ≥100 | 336 (90%) | 530 (90%) |
| ≥90<100 | 4 | 4 |
| ≥80<90 | 4 | 3 |
| ≥70<80 | 2 | 7 |
| ≥60<70 | 5 | 5 |
| ≥50<60 | 3 | 7 |
| ≥40<50 | 3 | 2 |
| ≥30<40 | 3 | 4 |
| ≥20<30 | 4 | 13 |
| ≥10<20 | 5 | 13 |
| <10 | 5 | – |
| Total | 374 (100%) | 588 (100%) |
| *Calculated magnetic field (mG)* | | |
| 0[a] | 323 (86%) | 510 (87%) |
| ≥0.001<0.1 | 35 (9%) | 57 (10%) |
| ≥0.1<0.2 | 4 | 9 |
| ≥0.2<0.3 | 3 | 4 |
| ≥0.3<0.4 | 2 | 2 |
| ≥0.4<0.5 | 4 | 1 |
| ≥0.5<0.6 | 0 | 0 |
| ≥0.6<0.7 | 2 | 0 |
| ≥0.7<0.8 | 0 | 0 |
| ≥0.8<0.9 | 0 | 1 |
| ≥0.9<1.0 | 0 | 0 |
| ≥1.0 | 1 | 4 |
| Total | 374 (100%) | 588 (100%) |

[a]See text for discussion of addresses in this cateogory.

**Table VI** Distance analysis, all data

| *Distance (m)* | *Cases (374)* | *Controls (588)* | *Odds ratio* | *95% confidence limits* |
|---|---|---|---|---|
| ≥100 | 336 | 530 | 1.00 | |
| <100 | 38 | 58 | 1.04 | 0.64–1.70 |
| ≥75<100 | 8 | 9 | 1.50 | 0.57–3.97 |
| ≥50<75 | 10 | 17 | 1.00 | 0.45–2.24 |
| ≥25<50 | 7 | 15 | 0.74 | 0.28–1.95 |
| <25 | 13 | 17 | 1.10 | 0.47–2.57 |

*Distance analysis*

Table VI examines the effect on case–control status of distance to the nearest overhead line up to 100 m, other overhead lines being ignored. There are no significantly raised odds ratios in any distance band and there is no evidence of any trend with distance. The 1.10 estimate for <25 m and the 0.74 estimate for 25–50 m are not significantly different from unity. The odds ratio for all addresses within 100 m is 1.04, with confidence limits between 0.64 and 1.70.

Tables VII and VIII present distance analyses for the data split in two ways. In each case, the reduced numbers of cases and controls lead to relatively wide confidence limits. The odds ratios for the OSCC/NRCT data (Table VII) are higher for every distance band, although the confidence limits are too wide to make any valid inferences of difference. The evidence from each source (and from both sources combined) is that odds ratios of between 0.69 and 1.59 are consistent with the data from any particular distance band, apart from the three cases and one control from the OSCC/NRCT data in the 75–100 m band, where the confidence limits are extremely wide.

Table VIII presents information for the two main morphology groups by distance. Again no significant differences emerge, and there is no trend with distance for either solid or non-solid tumours. The odds ratios lie generally between 0.70 and 1.32. Only the odds ratio of 2.59 for solid tumours in the 75–100 m distance band approaches statistical significance, but has a lower confidence limit of 0.72. There is no evidence for different effects of distance on solid or non-solid tumours.

*Magnetic-field analysis*

Relatively few case and control addresses achieved calculated magnetic fields greater than the assumed background level of 0.1 mG and in only five instances (one case and four controls) was the field strength greater than 1 mG. Table IX compares the odds ratios for magnetic field stratified in several ways. No relative risk estimate is significantly different from 1.0, and there is no trend with increasing magnetic field – there being, if anything, a suggestion of highest values at intermediate fields. Using four field categories, the category between 0.3 and 1.0 mG gives the highest estimate (2.60), but with 95% confidence limits of 0.75 and 9.04. The estimate for all fields greater than 0.1 mG is 1.19, but is still compatible with true risks of between 0.56 and 2.55.

**Table VII** Data source distance analysis

| | *Cases with one control each (OSCC/NRCT data)* | | | | *Cases with two controls each (Yorkshire Registry data)* | | | |
|---|---|---|---|---|---|---|---|---|
| *Distance (m)* | *Cases (160)* | *Controls (160)* | *Odds ratio* | *95% confidence limits* | *Cases (214)* | *Controls (428)* | *Odds ratio* | *95% confidence limits* |
| ≥100 | 142 | 146 | 1.00 | – | 194 | 384 | 1.00 | – |
| <100 | 18 | 14 | 1.44 | 0.62–3.38 | 20 | 44 | 0.86 | 0.48–1.62 |
| ≥75<100 | 3 | 1 | 3.40 | 0.33–34.53 | 5 | 8 | 1.18 | 0.38–3.67 |
| ≥50<75 | | 3 | 1.59 | 0.33–7.57 | 6 | 14 | 0.85 | 0.32–2.27 |
| ≥25<50 | 4 | 4 | 0.83 | 0.18–3.89 | 4 | 11 | 0.69 | 0.20–2.35 |
| <25 | 8 | 6 | 1.45 | 0.39–5.31 | 5 | 11 | 0.87 | 0.27–2.76 |

**Table VIII** Tumour morphology distance analysis

| | *Non-solid tumours* | | | | *Solid tumours* | | | |
|---|---|---|---|---|---|---|---|---|
| *Distance (m)* | *Cases (180)* | *Controls (277)* | *Odds ratio* | *95% confidence limits* | *Cases (194)* | *Controls (311)* | *Odds ratio* | *95% confidence limits* |
| ≥100 | 162 | 252 | 1.00 | – | 174 | 278 | 1.00 | – |
| <100 | 18 | 25 | 1.02 | 0.48–2.17 | 20 | 33 | 1.06 | 0.55–2.02 |
| ≥75<100 | 2 | 5 | 0.70 | 0.13–3.89 | 6 | 4 | 2.59 | 0.72–9.34 |
| ≥50<75 | 5 | 5 | 1.32 | 0.36–4.84 | 5 | 12 | 0.74 | 0.25–2.18 |
| ≥25<50 | 4 | 8 | 0.76 | 0.22–2.63 | 3 | 7 | 0.71 | 0.15–3.30 |
| <25 | 7 | 7 | 1.32 | 0.36–4.76 | 6 | 10 | 0.97 | 0.31–3.04 |

**Table IX** Magnetic-field analysis, all data

| *Magnetic field (mG)* | *Cases (374)* | | *Controls (588)* | | *Odds ratio* | | *95% confidence limits* | |
|---|---|---|---|---|---|---|---|---|
| ≤0.1 | 358 | | 567 | | 1.00 | | | |
| ≥0.1 | 16 | | 21 | | 1.19 | | 0.56–2.55 | |
| ≥0.1<1.0 | 15 | | 17 | | 1.35 | | 0.61–3.01 | |
| ≥1.0 | 1 | | 4 | | 0.39[a] | | 0.04–4.09 | |
| ≥0.1<0.3 | 7 | | 13 | | 0.96 | | 0.37–2.51 | |
| ≥0.3<1.0 | 8 | | 4 | | 2.60 | | 0.75–9.04 | |
| | | 9 | | 8 | | 1.73 | | 0.59–5.07 |
| ≥1.0 | 1 | | 4 | | 0.40[a] | | 0.04–4.33 | |

[a]These slightly different odds ratios for the ≥1.0 mG category are a consequence of case–control matching.

**Table X** Data source magnetic-field analysis

| | *Cases with one control each (OSCC/NRCT data)* | | | | *Cases with two controls each (Yorkshire Registry data)* | | | |
|---|---|---|---|---|---|---|---|---|
| *Magnetic field (mg)* | *Cases (160)* | *Controls (160)* | *Odds ratio* | *95% confidence limits* | *Cases (214)* | *Controls (428)* | *Odds ratio* | *95% confidence limits* |
| <0.1 | 151 | 154 | 1.00 | | 207 | 413 | 1.00 | |
| ≥0.1 | 9 | 6 | 1.60 | 0.52–4.89 | 7 | 15 | 0.90 | 0.31–2.67 |
| ≥0.1<1.0 | 9 | 5 | – | | 6 | 12 | 0.97 | 0.31–3.00 |
| ≥1.0 | 0 | 1 | – | | 1 | 3 | 0.59 | 0.05–7.50 |
| ≥0.1<0.3 | 3 | 4 | 0.86 | 0.19–3.96 | 4 | 9 | 0.90 | 0.26–3.09 |
| ≥0.3 | 6 | 2 | 2.95 | 0.59–14.75 | 3 | 6 | 0.96 | 0.19–4.88 |

**Table XI** Tumour morphology magnetic-field analysis

| | *Non-solid tumours* | | | | *Solid tumours* | | | |
|---|---|---|---|---|---|---|---|---|
| *Magnetic field (mg)* | *Cases (180)* | *Controls (277)* | *Odds ratio* | *95% confidence limits* | *Cases (194)* | *Controls (311)* | *Odds ratio* | *95% confidence limits* |
| <0.1 | 170 | 264 | 1.00 | | 188 | 303 | 1.00 | |
| ≥0.1 | 10 | 13 | 1.14 | 0.42–3.11 | 6 | 8 | 1.27 | 0.39–4.08 |
| ≥0.1<1.0 | 9 | 11 | 1.19 | 0.42–3.42 | 6 | 6 | – | |
| ≥1.0 | 1 | 2 | 0.82 | 0.07–9.64 | 0 | 2 | – | |
| ≥1.0<0.3 | 4 | 7 | 0.90 | 0.25–3.29 | 3 | 6 | 0.91 | 0.22–3.73 |
| ≥0.3 | 6 | 6 | 1.43 | 0.41–5.03 | 3 | 2 | 3.13 | 0.31–31.84 |

Tables X and XI split the data according to source and tumour morphology. As before, the OSCC/NRCT data (Table X) give higher odds ratios than the Yorkshire Registry data, but none of the estimates is significantly higher than unity. The confidence limits are particularly wide and uninformative for magnetic fields ≥1.0 mG.

Table XI does not give any indication that the different morphologies are affected differently by magnetic field. For both solid and non-solid tumours the highest odds ratios are for intermediate fields (between 0.1 and 1.0 mG). The highest estimate is for solid tumours (1.27), but the 95% confidence limits are between 0.39 and 4.08.

### *Possible confounding by house type*

Cases and controls had not originally been matched for house type, although it is a possible indicator of social class, which may be associated with the disease (Savitz *et al.*, 1988). Inspection of Table III reveals that cases were slightly more likely than controls to live in terraced houses.

Terraced houses were also less likely than semi-detached or detached to be within 100 m of overhead lines (7% compared with 12%, $P<0.05$) and less likely to have estimated line fields of more than 0.1 mG (3% compared with 5%, a non-significant difference). It is therefore conceivable that, in the overall analysis, a risk associated with magnetic fields or distance from lines could have been obscured by a separate relative risk associated with terraced housing.

An analysis restricted to case–control matches wholly within the terraced and semi-detached/detached housing categories, and ignoring any possible effect due to distance or magnetic field, gives an odds ratio for terraced houses of 1.14 (0.85–1.55).

Identical house types were shared by 234 of the 374 cases and 291 of the 588 matched controls. Among these 24 of the cases and 25 of the controls were within 100 m of overhead power lines, and 12 of the cases and seven of the controls were in an estimated magnetic field of ≥0.1 mG. This indicates that controlling for house type does not result in raised odds ratios for distance, but may affect our relative risk estimates for magnetic field. However, including 'house type' in a linear logistic model with either distance or magnetic field categories produced no significant estimates of relative risk.

In addition to the work on balanced phase currents presented here, we have investigated the possible effects of unbalanced phase currents. Such unbalance is normally appreciable for LV circuits only, but is extremely difficult to estimate with confidence.

In the absence of quantitative information for individual circuits, we made generalised estimates based on discussion with engineers of the YEB and NEEB. Incorporating these estimates into the analyses did not significantly change the results presented in this paper.

### Discussion

The above results do not support an association between the presence of childhood cancer and either the nearness or the calculated magnetic fields of overhead power lines. The field measurements which accompanied the study indicated that median domestic background fields due to sources other than the load currents of overhead lines were of order 0.1 mG. The case–control analysis presented shows no significant difference in odds ratio for calculated power line fields above or below this level; nor is there any convincing difference or trend in the odds ratios for different distances from the lines.

The few estimates of relative risk presented in this paper which approach statistical significance are either not part of a plausible trend, or are otherwise difficult to interpret. Thus, the highest estimate in Table IX is for an intermediate magnetic field strength.

The validity of allowing for a possible effect of house type as an indicator of social class may be questioned. Much of the terraced housing in the region is privately owned by the occupants, while much of the semi-detached housing is publicly owned and rented to the occupants. Furthermore, evidence from the literature that socioeconomic factors are associated with childhood cancers is limited.

By the very nature of this type of case–control study, the number of case and control children whose homes would be in different distance and magnetic field bands could not be known in advance. Thus, no statistical power estimations could be carried out *a priori*. However, with the wisdom of hindsight, an estimate can be made, based on the numbers of cases and controls whose birth addresses were within 25 m (30 children), and 100 m (approximately 100 children) of overhead power lines, or whose birth address had a calculated field of 0.1 mG or more (approximately 40 children) or 1.0 mG or more (five children).

Assuming a two-sided type I error of 5%, and independent samples, the study was calculated to have only an 18% power of detecting a true relative risk of 1.5 for those living within 25 m of an overhead line, or 54% power using 100 m as the critical distance. Acceptable statistical power is found either if a true relative risk more than 2.5 or 3 exists for children living within 50 m of power lines, or if a more moderate risk (2.0) occurs throughout the 0–100 m band.

If an above-background magnetic field carried a true relative risk of 2.5 or more, this study had a reasonable change of detecting it. The study stood no realistic chance of detecting any raised relative risk associated with a field of more than 1 mG, because of the very small numbers of cases and controls in that situation. Because many of the calculated fields were of the same order of magnitude as the assumed background field, and true background fields are likely to vary from place to place, the true statistical power of the study will be even less than the figures reported above.

In summary, then, because there were relatively small numbers of cases and controls in the 'exposed' categories, the study has a low statistical power of detecting quite moderate relative risks (2.0–2.5). The fact that significantly raised relative-risk estimates were not found may be due to the lack of a true association of risk with distance or magnetic field, or because the true relative risks are less than moderate, or because of a type I error. It is not possible to determine which of these is true.

Weaknesses of the study include the lack of any measurements of magnetic field at case or control addresses and the lack of consideration of a number of possible confounding factors in addition to house type (such as, for example, the mothers' exposure to X-rays during pregnancy). An important strength of the study is that the magnetic field estimates were based to a large extent on actual historical loads for the overhead lines concerned. However, the generally low level of calculated fields relative to the background from other sources means that the study reveals little about possible effects of magnetic fields *per se*.

The rarity of childhood cancer as a disease and the apparent rarity of enhanced exposure to magnetic fields produced by the load currents of overhead power lines means that future studies must be designed with greater numbers of cases and controls and/or with better characterisation of actual exposure to magnetic fields. Such studies are already underway in the United States and in Sweden and are planned for the UK in the near future.

We are grateful to many people who made this study possible, but especially to the engineers of the CEGB, the YEB and the NEEB, who provided maps and load data; to Jenny Jagucki and David Peters, who carried out the detailed mapping exercises; and to John Bonnell, Robin Cox, Brian Maddock and John Male of the CEGB, who provided much helpful comment and advice. The study was financed by the Central Electricity Generating Board and the Electricity Council, and the research was carried out by the University of Leeds. Thanks are also due to the Yorkshire Children's Cancer Registry, and for the support of the Oncology Research and Development Fund of the Leeds Western Health Authority Special Trustees.

### References

AHLBOM, A. (1988). A review of the epidemiologic literature on magnetic fields and cancer. *Scand. J. Work Environ. Health*, **14**, 337.

BRESLOW, N.E. & DAY, N.E. (1980). *Statistical Methods in Cancer Research, Vol. 1*. Oxford University Press: Oxford.

CARTWRIGHT, R.A. (1989). Low frequency alternating electromagnetic fields and leukaemia: the saga so far. *Br. J. Cancer*, **60**, 649.

DRAPER, G.J., BIRCH, J.M., BITHELL, J.F. & 6 others (1982). *Childhood Cancer in Britain*. Studies in Medical and Population Subjects, no. 37. HMSO: London.

FULTON, J.P., COBBS, S., PREBLE, L., LEONE, L. & FORMAN, E. (1980). Electrical wiring configurations and childhood leukaemia in Rhode Island. *Am. J. Epidemiol.*, **111**, 292.

MYERS, A., CARTWRIGHT, R.A., BONNELL, J.A., MALE, J.C. & CARTWRIGHT, S.C. (1985). Overhead powerlines and childhood cancer. International Conference on Electric and Magnetic Fields in Medicine and Biology. London 4–5 December.

ROTH, H.D. (1985). *An Evaluation of Published Studies Analysing the Association of Carcinogenesis with Exposure to Magnetic Fields*. Publication EA-3904. Electric Power Research Institute: Palo Alto, CA.

SAVITZ, D.A. (1986). Human health effects of extremely low frequency electromagnetic fields: critical review of clinical and epidemiological studies. Presented at IEEE Winter Power Meeting, New York City, 3 February.

SAVITZ, D.A., WACHTEL, H., BARNES, F.A., JOHN, E.M. & TVIRDIK, J.G. (1988). Case–control study of childhood cancer and exposure to 60-Hertz magnetic fields. *Am. J. Epidemiol.*, **128**, 21.

SAVITZ, D.A. & FEINGOLD, L. (1989). Assessment of childhood cancer with residential traffic density. *Scand. J. Work Environ. Health*, **15**, 360.

TOMENIUS, L. (1986). 50 Hz electromagnetic environment and the incidence of childhood tumours in Stockholm County. *Bioelectromagnetics*, **7**, 191.

WERTHEIMER, N. & LEEPER, E. (1979). Electrical wiring configurations and childhood cancer. *Am. J. Epidemiol.*, **109**, 273.

WERTHEIMER, N. & LEEPER, E. (1980). Electrical wiring configurations and childhood leukaemia in Rhode Island. *Am. J. Epidemiol.*, **111**, 461.